# Amplitude, relative phase, and polarization characterization during ultrashort pulse of hybrid mode-locked thulium-doped all-fiber laser

**DOI:** 10.1038/s41598-026-45251-4

**Published:** 2026-04-04

**Authors:** Daniil Batov, Vasilii Voropaev, Semyon Mizgirev, Vladimir Lazarev, Rick Trebino, Mikhail Tarabrin

**Affiliations:** 1 Science and Education Center for Photonics and IR-Technology, Bauman Moscow State Technical University, Moscow, 105005 Russia; 2 School of Physics, Georgia Institute of Technology, 837 State Street, Atlanta, GA 30332 USA

**Keywords:** Optics and photonics, Physics

## Abstract

Applications of ultrashort pulses at 1.9 ${\upmu }$m, such as micromachining, nonlinear imaging, and harmonic generation, require knowledge of the amplitude, phase, and polarization during the pulse. Therefore, the aim of this work is to characterize these parameters for pulses generated by a hybrid mode-locked thulium-doped fiber laser using the tomographic ultrafast reconstruction of transverse light fields (TURTLE) principle. The measurements are performed in two cases: directly at the laser output and after a polarization-independent isolator. Even at the laser output, the pulses exhibit a time-dependent polarization, confirming the intrinsic vector nature of nonlinear polarization rotation inherent in hybrid mode-locked lasers. Numerical simulations based on the coupled nonlinear Schrödinger equations reproduce this behavior qualitatively. Introducing a polarization-insensitive isolator significantly modifies the evolution of the pulse polarization due to uncompensated polarization mode dispersion, which splits the transmitted pulse into two pulses with opposite directions of electric field rotation. By using a polarization controller in front of the isolator, it is possible to adjust the power ratio between the two separated pulses, effectively suppressing the splitting process. These results demonstrate the importance of measuring and controlling the vectorial structure of ultrashort pulses in lasers based on non-polarization-maintaining fibers.

## Introduction

Ultrafast thulium-doped fiber lasers operating at 1.9 µm play an important role in applications such as micromachining^1^, nonlinear microscopy^2,^ and supercontinuum generation^3^. In some of these applications, not only the pulse duration and peak power but also the polarization state is crucial. For example, femtosecond-laser micromachining exhibits strong polarization-dependent ablation behavior, including symmetry changes and efficiency variations^4^, while polarization selectivity directly determines excitation efficiency in nonlinear microscopy^5^. These examples illustrate that characterization of the polarization of ultrashort pulses is often as important as measurements of temporal and spectral properties.

Studies of polarization dynamics in mode-locked fiber lasers have shown that birefringence can lead to the formation of various types of vector solitons, including polarization-locked and group-velocity-locked vector solitons^6–8^. These regimes have been primarily investigated in systems where mode locking is achieved using saturable absorbers. At the same time, in fiber laser systems, mode locking is often implemented using nonlinear polarization rotation (NPR) because of its simplicity. Due to the presence of a polarization-selective element inside the cavity, it is generally assumed that no vector solitons could be formed in the fiber lasers^8^. Nevertheless, NPR and birefringence of the fibers can lead to a change in polarization during the pulse. More than that, additional fiber-optic components placed after the cavity, such as isolators, may introduce polarization mode dispersion (PMD), which can alter the output polarization state in an uncontrolled manner^9^. In the sub-picosecond regime, even small differential group delays between orthogonal polarization components can lead to a nontrivial temporal evolution of the polarization state.

Despite the possible complex evolution of the polarization state, most experimental studies measure pulse characteristics either for a scalar projection (e.g., FROG or autocorrelation) or for the full polarization state of the radiation, averaged over the pulse in time^7,8^. Currently, several approaches exist that enable measurement of the polarization state profile within an ultrashort pulse. The first approach is interferometric and is based on determining the relative phase between two orthogonal polarization components using spectral interferometry (SI). This is achieved by measuring interference between projections of orthogonal components in either a single-channel configuration^10^ or a dual-channel configuration^11^. In these schemes, reconstruction of the pulse amplitude, phase, and polarization state requires the presence of a reference pulse with a known amplitude, phase, and polarization in one scalar projection. SI-based approaches offer high sensitivity, although they impose strict requirements on system stability and optical alignment. The second approach reconstructs the relative phase and delay from the amplitudes and phases of two orthogonal polarization components using an additional polarization projection. This approach forms the basis of techniques such as tomographic ultrafast reconstruction of transverse light fields (TURTLE)^12^, vectorial FROG^13^, and TURTLE based on d-scan measurements^14^. Since these methods are based on nonlinear processes (most often second harmonic generation (SHG)), they are generally less sensitive than methods based on SI but, at the same time, have a simpler design and require less time-consuming adjustment. The third approach, time-stretch-based polarimetry, is based on temporally stretching an ultrashort pulse by propagation through a dispersive medium and measuring the four Stokes parameters using relatively slow photodetectors^15^. This approach does not allow retrieval of the pulse amplitude and relative phase, but due to its sensitivity and possibility of real-time characterization, this technique is primarily used for studying polarization dynamics.

Although the aforementioned measurement approaches are capable of resolving polarization across ultrashort pulses, these techniques remain largely unused for characterizing pulses directly at the output of fiber laser systems. To date, only one study^16^ has demonstrated simultaneous measurements of the amplitude, phase, and polarization across the pulse in a fiber system, but that study was conducted after an amplification stage and did not investigate the output of the mode-locked laser itself. In our previous work^17^, we reported hybrid mode locking of a thulium-doped fiber laser employing single-walled carbon nanotubes (SWCNT) and NPR. However, only the output linear polarization and pulse duration were measured, while the underlying vector nature of the pulses remained unexplored. Characterizing the amplitude, phase, and polarization across the pulse is important for diagnosing polarization-dependent effects and designing fiber amplifiers where PMD can significantly distort the pulse.

Therefore, the goal of this work is to perform full vectorial characterization of ultrashort pulses generated by a hybrid mode-locked thulium-doped fiber laser. We chose TURTLE as the characterization method because it has proven effective in reconstructing the amplitude, phase, and polarization evolution of ultrashort pulses, and its operation only required the addition of a linear polarizer to the existing SHG-FROG setup in our laboratory^18,19^. TURTLE involves performing three FROG measurements of the pulse traces after the polarizer, extracting the vertical, horizontal, and $45^\circ$-projections. From the measured traces, the phase retrieval algorithm determines the complex envelope of the vertical and horizontal components. An additional algorithm then determines their relative phase and delay using the FROG trace measured for a $45^\circ$-projection. Numerical simulations based on coupled nonlinear Schrödinger equations (C-NLSEs) were also performed and time-dependent polarization evolution at the laser output was obtained. Finally, we investigate the effect of a polarization-independent isolator with uncompensated PMD on the pulse characteristics.

## Laser and experimental setup

**Figure 1:**
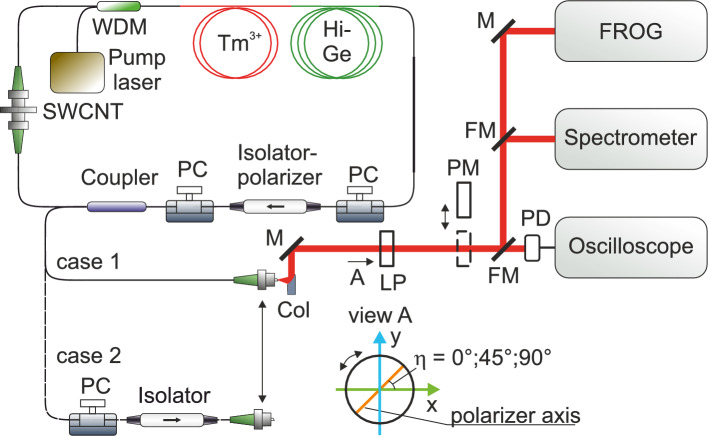
Schematic diagram of the thulium-doped fiber laser and experimental setup for measuring the evolution of the intensity, phase, and polarization state of the ultrashort laser pulse in time. WDM – wavelength-division multiplexer; $\hbox {Tm}^{3+}$ – thulium-doped fiber; Hi-Ge – fiber with high concentration of germanium oxide for dispersion compensation; SWCNT – single-walled carbon nanotubes; PC – polarization controller; Col – reflective collimator; M – mirror; FM – flip mirror; LP – linear polarizer; PD – photodetector; PM – power meter; FROG – frequency-resolved optical gating setup.

Figure 1 shows the schematic diagram of the ultrafast thulium-doped fiber laser as well as the experimental setup designed to measure the temporal evolution of the pulse intensity, phase, and polarization state. The laser setup and its numerical model are described in detail in Ref.^17^; here we briefly summarize the design and output parameters relevant to the measurements.

The laser uses hybrid mode locking, which is implemented using SWCNT^20,21^ and NPR^22,23^. The laser cavity consists of silica fibers that do not maintain polarization of the radiation. To compensate for the anomalous dispersion of standard fibers (SMF-28) and thulium-doped active fiber, fiber with a high concentration of germanium oxide in the core is used. To adjust the mode-locking regime in the laser, intracavity mechanical polarization controllers (PCs) CPC250 (Thorlabs Inc., USA) are used.

In the mode-locked regime, the laser radiation has the following parameters: center wavelength of about 1900 nm, spectral bandwidth of 22 nm, pulse duration of 300 fs, pulse repetition rate of 23.8 MHz, average power of about 6 mW, and pulse energy of 0.25 nJ. These parameters were measured without using a polarizer, so the polarization state was not measured. In the autocorrelator, the horizontal polarization of the radiation was measured due to the use of a crystal for SHG with type I phase matching.

In this paper, the full vector characterization of the ultrashort pulse of the laser under study is performed in two cases. In the first case, the radiation characteristics are measured directly at the laser output to determine the polarization characteristics of the radiation generated by the laser itself. In the second case, the radiation characteristics are measured at the output of a polarization-independent isolator (widely used in laser apparatuses). Polarization before the isolator can be adjusted using a PC. It is known that this type of isolator can introduce PMD and distort the pulse polarization, and the manufacturer may not always be able to provide PMD values.

In the measuring branch, the laser radiation is collimated using a reflective collimator RC02APC-P01 (Thorlabs, USA), passed through a linear polarizer LPMIR050-MP2 (Thorlabs, USA) mounted on a rotating frame with an angular scale, and directed to the measuring instruments using a system of metal plano mirrors either with unflip (Ms) or flip mounts (FMs). Metal-coated mirrors were chosen because metal, unlike dielectrics, typically does not perturb polarization. To measure the average power of radiation with orthogonal polarizations, a power meter (PM) 3A (Ophir, Israel) is used, which is introduced into the laser beam when desired. To control the generation regime, a high-speed PIN photodiode ET-5000F (Coherent, USA) with a bandwidth of more than 10 GHz is used, connected to an oscilloscope with a bandwidth of 1 GHz. To measure the intensity and phase of pulses over time (or complex spectral amplitudes), an SHG-FROG setup developed in the laboratory is used, based on a 600-µm-thick BBO crystal with type-I phase matching and antireflection coatings in the wavelength range from 1.8 to 2 µm on the input surface and from 0.9 to 1 µm on the output surface. The TURTLE principle is used to retrieve the evolution of the intensity, phase, and polarization state of the generated ultrashort pulse in time^12^. In TURTLE measurements based on SHG-FROG traces, a time direction (or handedness) ambiguity arises^12,24^. In our measurements, the time direction was not measured, as it was not the focus of this study. Nevertheless, the handedness ambiguity can be resolved using alternative FROG beam geometries, such as polarization-gate FROG or transient grating FROG, or in SHG-FROG by adding a known amount of material chirp in one or more of the SHG-FROG trace measurements^12,24^. The description of the TURTLE retrieval algorithm is given in Appendix A.

## Numerical and experimental results

For ease of reading, all measured and retrieved traces are provided in Appendix B, and the processed measurement results are presented below. Code for vectorial graph plotting using reconstructed complex field amplitudes in MATLAB and data files are provided in the Supplementary Materials. The Supplementary Materials also present video visualizations of the polarization ellipse rotation and electric field oscillations over time. The video visualizations on the right show the electric field projection ($E_x,E_y$), while the left shows the field oscillations in the $E_x$ projection over time. To understand the direction of polarization ellipse rotation, the video can be slowed down.

### Case 1: evolution of polarization, intensity and phase directly at the laser output

**Figure 2:**
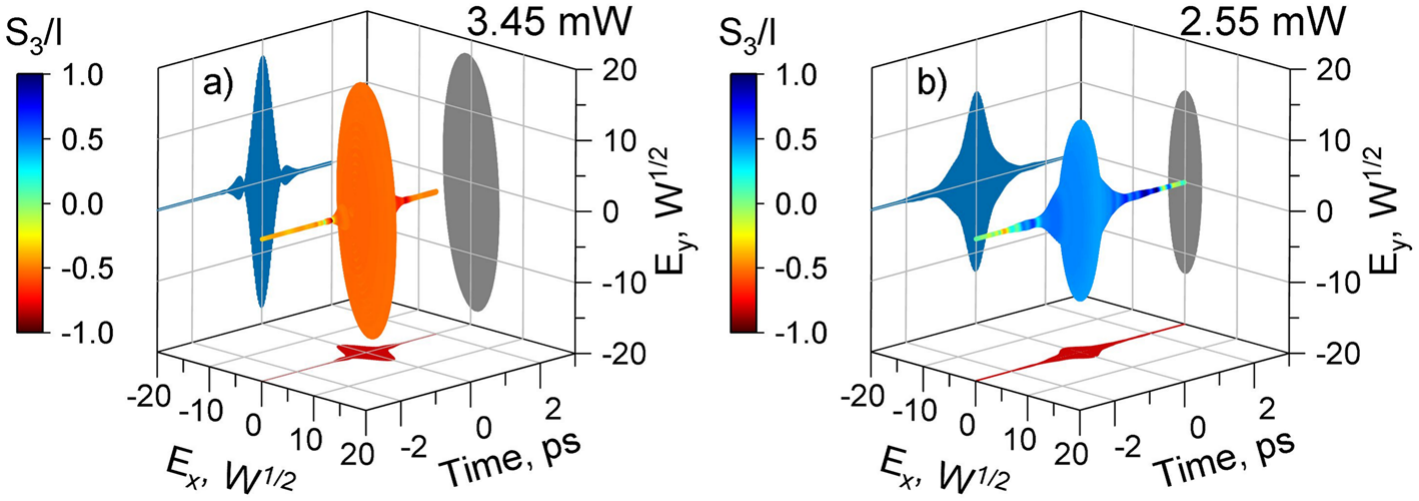
The simulated (**a**) and measured (**b**) vectorial pulses directly at the laser output. The color indicates the S3/I parameter, which characterizes the degree of circular polarization (-1 – left-handed circular polarization, 0 – linear polarization, 1 – right-handed circular polarization). See Visualization 1 and Data File 1 for the simulated vectorial pulse and Visualization 2 and Data File 2 for the measured one.

Figure 2 shows the simulated (a) and measured (b) evolution of the full electric field for the pulse directly at the laser output. The electric field is normalized such that the time integral over its envelope corresponds to the optical power^25^. The parameter $S_3/I$ is the third Stokes parameter, normalized to the full electric field intensity, it characterizes the degree of circular polarization: -1 refers to left-handed circular polarization, 0 to linear polarization and 1 to right-handed circular polarization. The figure also displays various projections of field evolution: $E_xE_y$, time-domain $E_x$, and time-domain $E_y$.

To better understand the possibility of generating pulses with complex evolution of polarization along the pulse in a hybrid mode locking scheme, we developed a numerical model. This model uses a split-step Fourier method to integrate C-NLSE with arbitrary linear birefringence and the rotation angle of the linear birefringence axes at each step in 1 mm increments, as described in detail in Ref.^26^. The average value of linear birefringence of the cavity is $0.75 \cdot 10^{-7}$. We extended this equation by taking into account the gain and absorption of fibers in the linear operator. The action of an SWCNT, coupler, and WDM is modeled in the same way as in Ref.^17^, except that the filter width of the WDM is 160 nm and does not limit the generation spectrum. PCs are described as a linear phase plate characterized by delay and rotation of the polarization axes. The effect of the isolator-polarizer is modeled by an ideal linear polarizer. Specifically, the field along one linear birefringence axis experiences attenuation equivalent to the polarization extinction ratio (PER) for the polarizer, while the field along the other axis passes through the polarizer without loss. When setting up the PCs in the numerical model, we obtained many different regimes at the laser output, differing in polarization ellipses; here we present the one whose average PER was closest to the measured one.

As can be seen from Fig. 2a,b, the output polarization of the laser in both the simulations and experiments is elliptical, exhibiting unidirectional rotation, with the $S_3/I$ varying from -0.50 to -0.43 for the simulation and from 0.43 to 0.47 for the measurements in the range of durations corresponding to half the maximum of the total pulse intensity. At the pulse fronts, the polarization becomes closer to circular for the simulated ($S_3/I$ down to -0.52) and measured ($S_3/I$ up to 0.53) pulses. Both numerical simulations and measurements yielded pulses with predominantly elliptical polarization at the pulse temporal center, becoming more circular at the leading edges. The directions of electric field rotation are different for simulated and measured pulses. The average power in the simulation was 3.45 mW, while the measured power was 2.55 mW. The discrepancies between the model and experiment are explained in the Discussion section.

**Figure 3:**
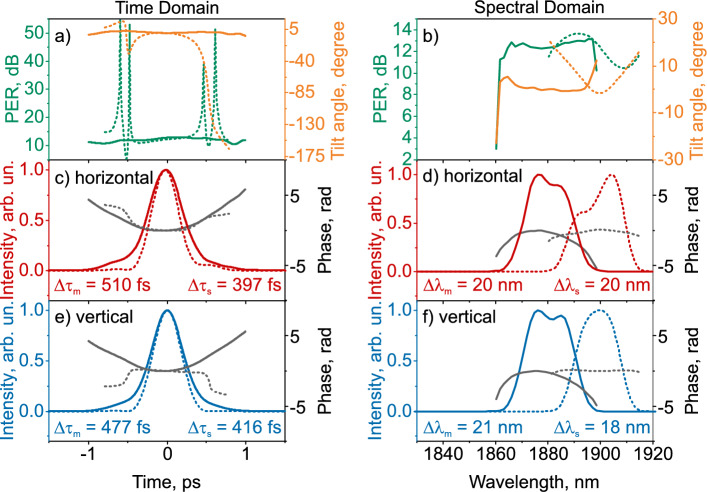
Simulated (dashed line) and measured (solid line) characteristics of pulse at the laser output in time (**a**,**c**,**e**) and spectral (**b**,**d**,**f**) domains: (**a**,**b**)—polarization extinction ratio (PER) and polarization ellipse tilt angle between the vertical axis and the major axis of the polarization ellipse, (**c**,**d**) and (**e**,**f**)—intensity and phase for the horizontal and vertical planes of pulse polarization, respectively.

Figure 3a shows the pulse PER in the time domain, defined as $10 \lg \left( P_{\text{ maj }} / P_{\text{ min }}\right)$, where $P_{\text{ maj }}$ and $P_{\text{ min }}$ are the powers along the ellipse’s major and minor axes, respectively. The measured PER in the time (Fig. 3a) and spectral (Fig. 3b) domain varies slightly, with an average value of 12.2 dB for pulse intensities greater than $1/e^2$ of the maximum (solid green lines). The simulated PER near the central part of the pulse in the time domain varies slightly, with an average value of 11.2 dB (green dotted line). At the edges of the pulse, the PER varies significantly over time, corresponding to two lateral subpulses. In the spectral domain, the simulated PER varies from 10 dB to 14 dB. The angle between the vertical axis and the principal axis of the ellipse–the tilt angle–changes symmetrically relative to the central part of the pulse: within $1.5^\circ$ in the measured case and $7 ^\circ$ in the simulated case, which is a manifestation of NPR: low-intensity components have different tilt angles of the ellipse compared to high-intensity components. The tilt angle of the polarization ellipse in the spectral region for the measured case varies within $4^\circ$, for the simulated case within $12^\circ$.

The simulated and measured temporal and spectral intensity and phase of the orthogonal components are shown in Fig. 3c,e and d,f, respectively. As shown in Fig. 3c,e, the pulse has an up-chirp, and the phase slope for the simulated pulse is close to the measured one. The durations of the central part in orthogonal polarizations for the measured and simulated cases differ by 33 fs and 19 fs, respectively. The bandwidths in orthogonal polarizations for the measured and simulated cases differ by 1 nm and 2 nm, respectively.

Thus, simulated and measured features of polarization evolution at the fiber laser output were obtained. These features are primarily related to the action of NPR.

### Case 2: evolution of polarization, intensity and phase with polarization-independent isolator and additional polarization controller at the laser output

**Figure 4:**
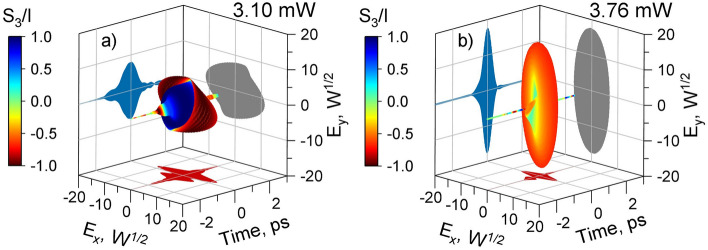
The reconstructed vectorial pulses with complex (**a**) and simple (**b**) polarization evolution after the isolator for two different additional PC adjustments. The color indicates the S3/I parameter, which characterizes the degree of circular polarization (-1 – left-handed circular polarization, 0 – linear polarization, 1 – right-handed circular polarization). For complex polarization evolution, see Visualization 3 and Data File 3; for simple, Visualization 4 and Data File 4.

Figure 4 shows a polarization evolution measurement for case 2 with a polarization-independent isolator at the output and various settings of an additional PC before the isolator. By tuning the PC settings, two characteristic polarization evolutions were obtained: complex (Fig. 4a) and simple (Fig. 4b). When setting up a PC, complex polarization evolution is more common than simple polarization evolution. This is likely due to the need to simultaneously meet two conditions for simple polarization. First, the PER at the isolator input must be sufficiently high, approximately greater than 10 dB. Second, the principal axis of the polarization ellipse at the isolator input must coincide with one of the axes of the isolator’s birefringent crystal. To quickly assess the evolution of the pulse polarization, we measured the spectral power densities in orthogonal polarizations using an OSA207C Fourier spectrometer (Thorlabs, USA). In the complex case, the spectral power densities clearly showed two peaks with different power ratios in the two orthogonal polarizations. In the simple case, the shapes of the spectral power densities for the orthogonal polarizations differed only slightly. The results of the spectral measurements are presented in the Appendix B Case 2.

In the complex polarization evolution (Fig. 4a), two sub-pulses with opposite field rotation directions were observed. The energy ratio between two sub-pulses with opposite field rotation directions was 67% and 33%, respectively. The delay between two orthogonal polarization peaks was 565 fs, which is related to the pulse duration and the manufacturer’s stated PMD (approximately 200 fs). The average power in the complex case was 3.10 mW.

In the simple polarization evolution (Fig. 4b), the pulse shape resembled that obtained directly at the laser output. The energy ratio between the elliptical polarizations rotating in opposite directions was 98% (left-handed) and 2% (right-handed), respectively. The delay between the two main peaks of orthogonal polarization for the simple polarization evolution is 5 fs. The average power in the simple case was 3.76 mW.

**Figure 5:**
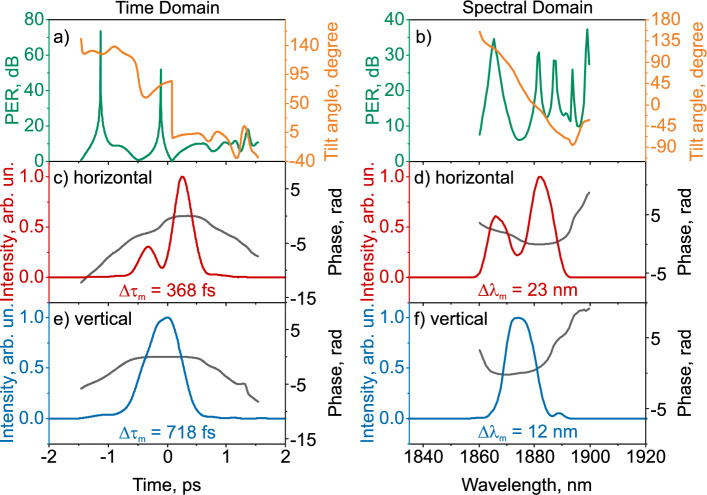
Temporal (**a**,**c**,**e**) and spectral (**b**,**d**,**f**) measured characteristics of the laser output radiation with an additional polarization controller and a polarization-independent isolator for the case of complex polarization evolution: (**a**,**b**)—polarization extinction ratio (PER) and polarization ellipse tilt angles, (**c**,**d**) and (**e**,**f**)—intensity and phase for the horizontal and vertical planes of pulse polarization, respectively.

Figure 5 shows the temporal (a, c, e) and spectral (b, d, f) characteristics of the polarization ellipse, and the intensity and phase in orthogonal polarizations in the case of complex polarization evolution.

In the time domain, the evolution of the polarization ellipse significantly changes both the PER and the tilt angle. The PER in the time domain varies from 0 dB to 52 dB and back, which corresponds to a twofold change in circular polarization, with a difference of 566 fs. The tilt angle changes quite steeply at the moment when polarization transitions from circular to linear, by $87^\circ$. In the spectral domain, PER fluctuates between 6.1 dB and 34 dB. The polarization-ellipse tilt evolves smoothly by $108^\circ$ at the half-maximum level of the spectral intensity.

The temporal and spectral intensity and phase of the orthogonal components are shown in Fig. 5c,e and d,f, respectively. In the horizontal polarization projection (Fig. 5c) two sub-pulses are observed, separated by an interval of 565 fs, while in the vertical polarization projection one pulse is observed. The spectral intensity of the pulse in the horizontal polarization projection has two peaks separated by 15 nm, while in the vertical polarization projection one peak is observed. Complex polarization evolution pulses have the temporal phase down-chirp. The durations in orthogonal polarizations differ by 350 fs, while the bandwidth differ by 11 nm.

As the measurement results show, adding a polarization-insensitive isolator to the mode-locked laser output significantly alters the evolution of the polarization ellipse. The PER value and the ellipse tilt angle change during the pulse, demonstrating both linear and circular polarization at different field rotations. The spectral width and pulse duration for orthogonal polarization differ by almost a factor of 2.

**Figure 6:**
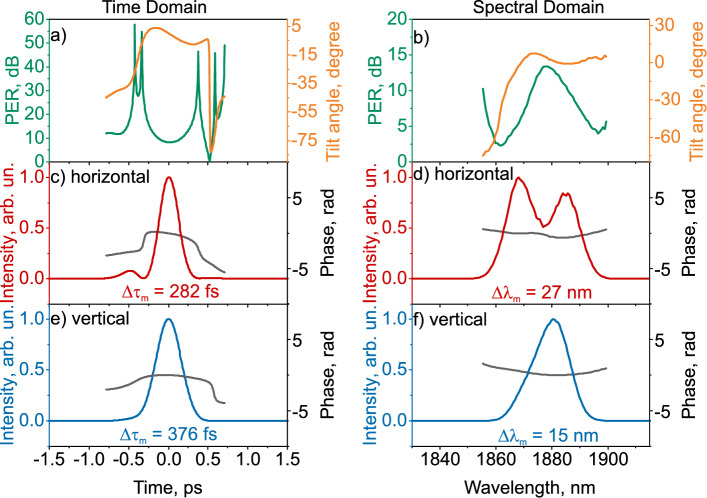
Temporal (**a**,**c**,**e**) and spectral (**b**,**d**,**f**) measured characteristics of the laser output radiation with an additional polarization controller and a polarization-independent isolator for the case of simple polarization evolution: (**a**,**b**)—polarization extinction ratio (PER) and polarization ellipse tilt angles, (**c**,**d**) and (**e**,**f**)—intensity and phase for the horizontal and vertical planes of pulse polarization, respectively.

Figure 6 shows the temporal (a, c, e) and spectral (b, d, f) characteristics of the polarization ellipse, and the intensity and phase in orthogonal polarizations in the case of simple polarization evolution.

In contrast to the complex evolution of polarization, in the simple case the polarization along the pulse does not change significantly. The PER in the time domain increases smoothly and symmetrically from 8.3 dB at the maximum to 10.6 dB at half maximum intensity. The tilt angle also changes slightly over time, by only about $6^\circ$. In the spectral domain, PER decreases symmetrically from 13.3 dB at maximum pulse intensity to 7.5 dB at a level corresponding to half the spectral pulse intensity. The tilt angle of the ellipse in the spectral domain changes by only $11^\circ$.

The temporal and spectral intensity and phase of the orthogonal components are shown in Fig. 6c,e and d,f, respectively. According to Fig 6c, in the horizontal polarization plane near the main pulse is a small intensity sub-pulse separated from the main peak by 488 fs. The spectral shape of the pulse in the horizontal polarization projection has two peaks separated by 16 nm, while in the vertical polarization projection one peak is observed. Simple polarization evolution pulses have the temporal phase down-chirp. The pulse durations of the orthogonal components differ by 94 fs, and their bandwidths differ by 12 nm.

As the measurement results show, adding a PC to the output of a laser with a polarization-insensitive isolator can prevent pulse splitting. In the simple case, the PER value is high, as is the case with the laser output (Fig. 3a), and the polarization ellipse tilt angle remains virtually unchanged. The duration and bandwidth of pulses in orthogonal polarizations differ, with the bandwidth of the pulse differing by almost two times.

## Discussion

This section discusses the variability of generation regimes depending on the PC settings in both the experiment and simulation, as well as possible future research directions. We needed to adjust the PCs in each measured case to initiate mode locking. This is probably due to changes in the induced birefringence of the cavity under the influence of external factors (temperature, pressure, and humidity), since each measurement took place on different days. This, in turn, resulted in different laser output parameters. The average output power varied in the range from 2 to 6 mW (Figs. 2b, 4a,b, Ref.^17^). Depending on the measured projection, the central wavelength varies from 1880 to 1900 nm, and the pulse duration varies from 282 to 718 fs (Figs. 6c, 5e). Furthermore, the numerical simulation results (average power, central wavelength, and pulse durations in orthogonal polarizations) also vary in a manner similar to the experiment with different PC settings. The magnitude of birefringence in the cavity fibers and the birefringence introduced by PCs are unknown. Therefore, only a qualitative comparison of the numerical simulation results and experimental data is possible.

In the paper^16^, a complex polarization state vs. time was demonstrated along the pulse at the output of a fiber-optic amplifier. In our work, on the other hand, the vector structure of the pulse is already present at the laser output. Therefore, a natural continuation of this work would be to investigate the polarization along the pulse after a thulium fiber amplifier^19^, where higher optical powers, stronger heating, and temperature-dependent phase delays in fiber components may lead to even more pronounced polarization distortions.

## Conclusion

We have experimentally demonstrated the full vectorial characterization of ultrashort pulses generated by a hybrid mode-locked thulium-doped fiber laser at 1.9 µm using the TURTLE principle. Measurements show that even at the laser output, the polarization state of the electric field changes along the pulse, which confirms the inherent vector nature of NPR-based mode locking. This behavior is qualitatively reproduced by C-NLSE simulations. Adding a polarization-insensitive isolator at the output of a mode-locked laser can lead to pulse splitting into two components rotating in opposite directions, as observed in the complex case. Placing a PC before the polarization-insensitive isolator prevents such splitting, as in the simple case. The simple polarization evolution is likely caused by the rotation of the polarization ellipse such that its principal axis aligns with one of the axes of the isolator’s birefringent crystal. In the complex case, however, the principal axis of the polarization ellipse at the isolator input does not align with either axis of the birefringent crystal. Therefore, when characterizing mode-locked fiber lasers, random angles between the polarization ellipse axis and the crystal axis can result in different evolutions of polarization, intensity, and phase of the output pulse. These findings highlight the importance of accounting for time-dependent polarization evolution in the design of non-polarization maintaining fiber lasers and amplifiers.

## Supplementary Information

Supplementary Information.

## Figures and Tables

**Figure 7:**
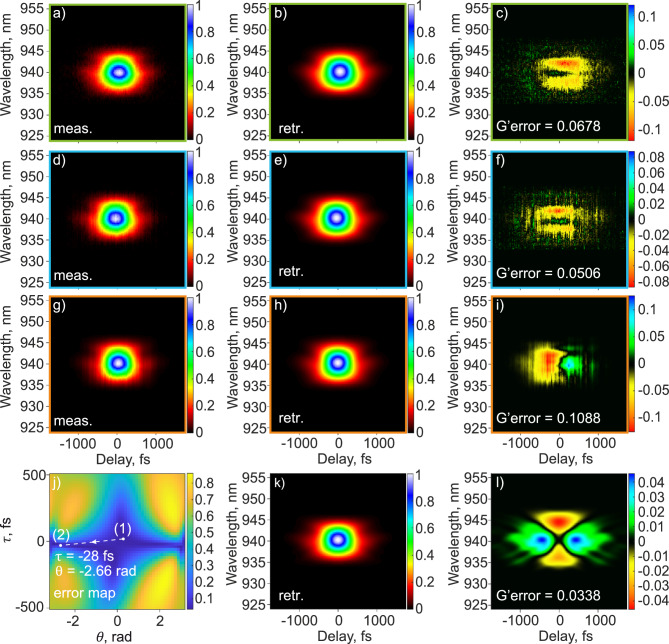
Measured, retrieved FROG traces, and the difference between measured and retrieved traces for $0^{\circ }$-projection (**a**–**c**), $90^{\circ }$-projection (**d**–**f**), and $45^{\circ }$-projection (**g**–**i**) for case 1. Map of $G'$ errors between retrieved $45^{\circ }$-projection and obtained using Eq. (2) trace (**j**), obtained trace after local optimization (**k**), difference between the (**h**) and (**k**) traces (**l**). Point (1) indicates the minimum in the global map, point (2) indicates the minimum found using local optimization.

**Figure 8:**
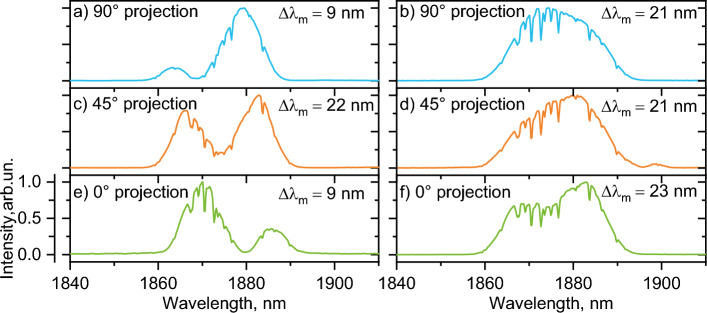
Measured spectrum for three polarization projections: $90^{\circ }$, $45^{\circ }$, and $0^{\circ }$ of the pulse for case 2 with complex (**a**,**c**,**e**) and simple (**b**,**d**,**f**) polarization evolution.

**Figure 9:**
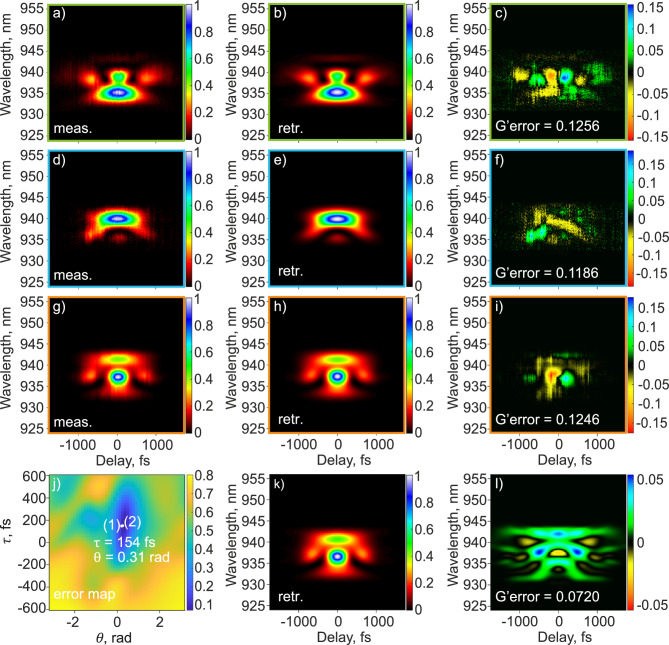
Measured, retrieved FROG traces, and the difference between measured and retrieved traces for $0^{\circ }$-projection (a,b,c), $90^{\circ }$-projection (d,e,f), and $45^{\circ }$-projection (g,h,i) for case 2, complex polarization evolution. Map of $G'$ errors between retrieved $45^{\circ }$-projection and obtained using Eq. (2) trace (j), obtained trace after local optimization (k), difference between the (h) and (k) traces (l). Point (1) indicates the minimum in the global map, point (2) indicates the minimum found using local optimization.

**Figure 10:**
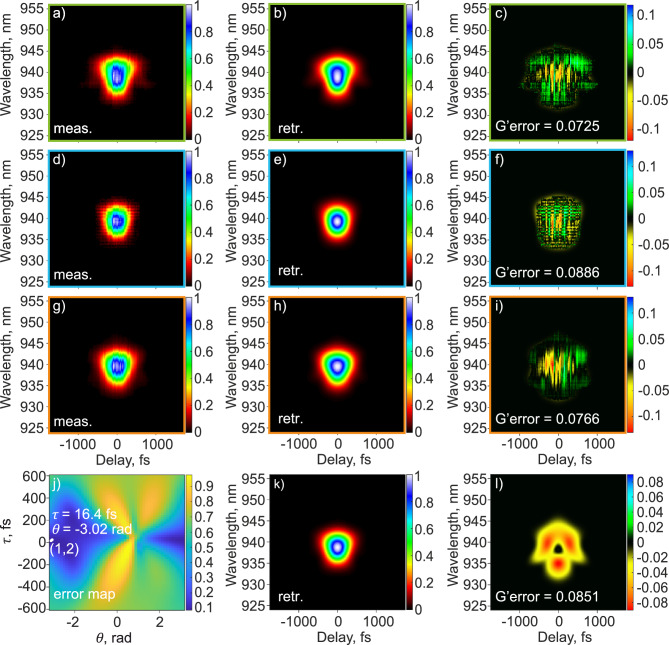
Measured, retrieved FROG traces, and the difference between measured and retrieved traces for $0^{\circ }$-projection (**a**–**c**), $90^{\circ }$-projection (**d**–**f**), and $45^{\circ }$-projection (**g**–**i**) for case 2, simple polarization evolution. Map of $G'$ errors between retrieved $45^{\circ }$ projection and obtained using Eq. (2) trace (**j**), obtained trace after local optimization (**k**), difference between the (**h**) and (**k**) traces (**l**). Point (1) indicates the minimum in the global map, point (2) indicates the minimum found using local optimization.

## Data Availability

Data underlying the results presented in this paper is not publicly available at this time but may be obtained from the authors upon reasonable request.

## Appendix A: TURTLE retrieval approach

The main goal of the TURTLE retrieval procedure is to find the relative phase $\theta$ and the relative delay $\tau$ between $0^{\circ }$ and $90^{\circ }$ pulse projections ($\tilde{E}_{x}$ and $\tilde{E}_{y}$, respectively)^12^. To determine $\theta$ and $\tau$ between the two projections, we measure three FROG traces for $0^{\circ }$, $45^{\circ }$, and $90^{\circ }$ projections and the ratio of the average powers of pulses with orthogonal polarizations $r = \sqrt{P_y/P_x}$.

In all experiments, the measured FROG traces had 350 delay points with a step of 20.01 fs and 501 wavelength points with a step of 0.06 nm, with a central wavelength of 935 nm. Before the retrieving procedure, we increased the FROG trace size to 4096x4096 points using linear interpolation and extrapolated the out-of-trace region with zeros as we did previously^18^ to increase the spectral and temporal resolution of the spectrogram input data for the retrieving algorithm. Thus, each FROG trace before the retrieving procedure has a temporal width of 20521 fs with a step size of 5.01 fs and a spectral width of 4.197 THz with a step size of 48.73 GHz (0.14 nm). For the retrieving algorithm, we used the RANA approach^27^, which combines the generalized projections algorithm with multiple grids and accurate initial guesses for the spectrum determined directly from the SHG FROG trace.

After the reconstruction procedure we get complex spectral amplitudes of the pulses with orthogonal polarizations $\tilde{E}_{x}(\Omega )$, $\tilde{E}_{y}(\Omega )$. We create a set of the relative phase and delay $(\tau , \theta )$. The temporal range is [-500 fs, 500 fs] with the step of $\delta \tau = 20$ fs, and the phase range is $[-\pi$ rad, $\pi$ rad) with the step of $\delta \theta = \pi /25$ rad. We calculate the set of complex spectral amplitudes of a field with polarization in a plane at $45^{\circ }$ using:

1 $$\begin{aligned} \tilde{E}_{\eta }(\Omega )=\cos {\eta } \tilde{E}_{x}(\Omega )+r\sin (\eta )\tilde{E}_{y}(\Omega )e^{-i(\Omega \tau +\theta )}. \end{aligned}$$

For each relative phase and delay $(\tau , \theta )$ we find $\textrm{E}_{\eta }(t)$ from $\tilde{E}_{\eta }(\Omega )$ using Fourier transform and calculate the SHG-FROG trace using the following expression:

2 $$\begin{aligned} I_{\textrm{calc}}(\omega , \tau )=\left| \int _{-\infty }^{\infty } \textrm{E}_{\eta }(t)\textrm{E}_{\eta }(t-\tau ) \exp (-\textrm{i} \omega t) d t\right| ^2. \end{aligned}$$

The $G'$ error between the retrieved and calculated SHG traces for each point in the set was calculated using the expression:

3 $$\begin{aligned} G^{\prime }=\sqrt{\sum _{i=1}^N \sum _{j=1}^N\left[ \frac{I_{\text{ retr } }\left( \omega _i, \tau _j\right) -\mu I_{\text{ calc }}\left( \omega _i, \tau _j\right) }{I_{\text{ retr }}\left( \omega _i, \tau _j\right) }\right] ^2}, \end{aligned}$$

where $I_{\text{ calc }}$ – is the calculated FROG trace, $I_{\text{ retr }}$ – the retrieved FROG trace, $\mu$ – the optimal scaling factor obtained from the error minimization criterion, *N⨯N* – the dimension of the FROG trace, and $\omega _{i}$ and $\tau _{j}$ are the *i*-th frequency and *j*-th delay in the frequency and delay vectors, respectively. It should be noted that when we obtain complex amplitudes ${E}_{x}(t)$, ${E}_{y}(t)$ from the measured FROG traces, we also calculate the reconstruction error, except that, in the Eq. (3), $I_{\text{ retr }}$ is replaced by $I_{\text{ meas }}$, and $I_{\text{ calc }}$ by $I_{\text{ retr }}$. It should also be noted that to obtain a perfectly symmetric $45^{\circ }$-projected SHG-trace, we retrieve the trace for $45^{\circ }$-projection, although this is not necessary^12^.

In the map of $G'$ errors between the retrieved and calculated SHG traces, we find the minimum point, which we then use as an initial guess for local two-parameter $(\tau , \theta )$ optimization. Local two-parameter optimization is implemented based on the Nelder-Mead simplex method. The minimum of local optimization corresponds to the found values of $(\tau , \theta )$.

Our TURTLE measurements reliably retrieved the relative temporal delay and phase between the two orthogonal polarization components, confirming the accuracy of the full vectorial reconstruction. We retrieve with two relative time directions: for $\tilde{E}_{x}(\Omega )$ and $\tilde{E}_{y}(\Omega )$ and for $\tilde{E}_{x}(\Omega )$ and $\tilde{E}_{y}^{*}(\Omega )$. For all TURTLE retrievals, the errors between the measured and retrieved traces were approximately twice as large for one of the time-direction choices. This confirms that the relative time direction between the two polarization projections is correctly retrieved. The absolute time direction was not measured, as we focus on the variation of polarization rather than its sign.

## Appendix B: Results

### Case 1: Evolution for the laser scheme without polarization-independent isolator

The measured, retrieved, and difference between measured and retrieved FROG traces for the laser scheme without a polarization-independent isolator (case 1) are shown for $0^{\circ }$-projection (a,b,c), for $45^{\circ }$-projection (d,e,f), for $90^{\circ }$-projection (g,h,i) in Fig. 7. The measured SHG-FROG traces corresponding to the $0^{\circ }$, $90^{\circ }$, and $45^{\circ }$ polarization projections are nearly identical. As stated above, the pulse mainly has elliptical polarization during the pulse.

Figure 7j shows $G^{\prime }$ errors between the retrieved trace (Fig. 7h) and the trace calculated using Eq. (2). Figure 7k shows the trace with minimum $G^{\prime }$ error after Nelder-Mead optimization, and Fig. 7l shows the difference between the calculated trace with minimum $G^{\prime }$ error (Fig. 7k) and the retrieved trace (Fig. 7h). The value for $G^{\prime }$ indicates excellent convergence.

### Case 2: Evolution for the laser scheme with polarization-independent isolator and polarization controller

As discussed in the main text, adding a polarization-independent isolator and a PC at the laser output allows for both complex and simple pulse polarization evolutions to be realized. For complex pulse polarization evolution, when adjusting the additional PC at the laser output, a two-peak structure is formed in the spectrum with different energy distributions between the two peaks in orthogonal polarizations (Fig. 8).

The measured, retrieved, and difference between measured and retrieved FROG traces for the laser scheme with polarization-independent isolator (case 2, complex polarization evolution) are shown for $0^{\circ }$-projection (a,b,c), for $90^{\circ }$-projection (d,e,f), and for $45^{\circ }$-projection (g,h,i) in Fig. 9.

Figure 9j shows $G^{\prime }$ errors between the retrieved trace (Fig. 9h) and the trace calculated using Eq. (2). Figure 9k shows the trace with minimum $G^{\prime }$ error after optimization, and Fig. 9l shows the difference between the calculated trace with minimum $G^{\prime }$ error (Fig. 9k) and the retrieved trace (Fig. 9h).

For the case of simple evolution of pulse polarization, the distribution of the spectral power density in orthogonal polarizations changed less than in the case of complex polarization (Fig. 8). The measured, retrieved, and difference between measured and retrieved FROG traces for the laser scheme with polarization-independent isolator in the case of simple polarization evolution (case 2, simple polarization evolution) are shown for $0^{\circ }$-projection (a,b,c), for $90^{\circ }$-projection (d,e,f), and for $45^{\circ }$-projection (g,h,i) in Fig. 10. As in Case 1, the SHG-FROG traces for the $0^{\circ }$, $90^{\circ }$, and $45^{\circ }$ polarization projections are nearly identical. Figure 10j shows $G^{\prime }$ errors between the retrieved trace (Fig. 10b) and calculated using Eq. (2). Figure 10k shows the trace with minimum $G^{\prime }$ error after optimization, and Fig. 10l shows the difference between the calculated trace with minimum $G^{\prime }$ error (Fig. 10k) and the retrieved trace (Fig. 10h).
